# Emotion-focused therapy in the treatment of prolonged grief disorder: study protocol for a randomized controlled trial

**DOI:** 10.1186/s13063-026-10005-0

**Published:** 2026-09-21

**Authors:** José Gamoneda, José Manuel Caperos, Pablo F. Cáncer, Rafael Jódar

**Affiliations:** Clinical Psychology Unit (UNINPSI), Department of Psychology, Faculty of Human and Social Sciences, Comillas Pontifical University, Street Universidad de Comillas 3-5, Madrid, 28049 Spain

**Keywords:** Grief, Bereavement, Grief with complications, Emotion-focused therapy, Prolonged grief disorder, PGD, Randomized controlled trial, RCT, Treatment effectiveness

## Abstract

**Background:**

Prolonged grief disorder (PGD) impacts various dimensions of the bereaved person’s life, such as psychological, social, and/or health domains. Psychological interventions, particularly those beyond the cognitive-behavioral approach, are essential to address these difficulties. This is especially relevant given that not all interventions are effective for all bereaved individuals, leaving a subset of people without adequate help. Emotion-Focused therapy (EFT) offers a promising alternative to support individuals suffering from PGD.

**Methods/design:**

This protocol describes a two-arm randomized controlled trial designed to evaluate the efficacy of EFT compared to a self-help control group for grief complications. The study will recruit 128 adults who meet eligibility criteria through the Clinical Psychology Unit of Pontifical Comillas University (UNINPSI) and the university’s networks. Participants will be randomly assigned to one of the two conditions. The EFT condition will include between eight and ten face-to-face weekly sessions. The self-help control group will consist of ten sessions of a self-help intervention based on EFT. Grief symptoms, anxiety, depression, trauma, and psychological distress will be measured using validated instruments (e.g., PG-13-R or CORE-10) before the intervention, during (at 3 weeks after the start), at the end of the intervention (at 12 weeks after the start, post-treatment), at 5 months, and at 12 months after the beginning of the intervention as follow-up assessments. In addition, grief and psychological distress will be assessed in all sessions (using the PG-13-R and CORE-10), and the therapeutic alliance will be measured in sessions 3 and 8 (using the WAI-S). A pilot study will precede the trial in order to adjust procedures and ensure adherence to the assessment protocol.

**Discussion:**

This study aims to establish the feasibility and comparative efficacy of EFT versus the self-help control group for PGD, hypothesizing that the EFT condition will demonstrate superior effectiveness at the different assessment points. The findings will expand the evidence base on therapeutic approaches to effectively support individuals with PGD.

**Trial registrations:**

ClinicalTrials.gov NCT07207512. Registered on 3 October 2025.

**Supplementary Information:**

The online version contains supplementary material available at https://doi.org/10.1186/s13063-026-10005-0.

## Background

Grief is a natural response to loss, a process that facilitates an individual’s adaptation to life after the death of a loved one [1]. In general, most bereaved individuals are able to process the loss adequately [2]. However, a minority of grievers experience difficulties throughout this process, developing what is termed prolonged grief disorder (PGD) [3], which affects their well-being across various domains, including health, social, and psychological areas [4]. This is particularly the case when certain risk factors are present, such as perinatal loss [5].

Precisely those individuals who experience PGD or are at risk of developing it are the ones who benefit most from psychological interventions [6]. However, as noted in the literature, not all interventions are effective for all bereaved individuals [7]. Adding to this, the fact that most grief interventions have a cognitive-behavioral basis highlights the need to test new therapeutic techniques and interventions. Therapies such as Emotion-Focused Therapy (EFT) have some preliminary evidence but require further research to be established as an option for treating PGD [8]. Although initial findings are encouraging, the empirical base remains limited, as no randomized controlled trial has yet examined EFT for PGD. It is therefore essential to provide alternatives that address the diversity and complexity of grief processes, with the aim of offering more effective and heterogeneous support to help bereaved individuals experiencing complications. This need becomes even more pressing considering that, in 2023, an estimated 254,699 people in Spain may have experienced complications in their grieving process, according to the grief index [9], which was calculated based on a prevalence of 9.8% for PGD [10] and mortality data from the Spanish National Statistics Institute (INE) for that year [11]. However, despite these alarming figures, the actual number may be underestimated, as it does not include individuals who were already suffering from this disorder. Furthermore, considering that at the outpatient level it has been estimated that 30% of patients referred to mental health services present grief complications [12], the need to attend to these individuals becomes evident.

EFT is a neo-humanistic therapy that synthesizes the person-centered tradition with a focus on experience. In this way, it aims at emotional awareness, expression, and regulation, as well as the creation of meaning from emotional experience and the transformation of problematic core emotions through change by means of emotion, which constitutes its main objective [13, 14]. This therapy has gathered evidence across various psychological difficulties such as depression [15], generalized anxiety [16], or interpersonal problems and trauma [17, 18]. Given its focus on the transformation and processing of problematic emotions, EFT may be a suitable choice for addressing grief complications. For all these reasons, this project aims to test EFT in the treatment of PGD.

### Objectives

The overall objective is the design of a two-arm randomized controlled trial (RCT) to establish the superior efficacy of the EFT group compared to the self-help control group. The main hypothesis is that the intervention delivered within the EFT model will demonstrate greater effectiveness during the intervention (at 3 weeks after the start of treatment), at post-treatment (12 weeks after the start of treatment), and at follow-up (5 and 12 months after the start of treatment). Secondarily, we expect that improvement in the EFT group will be greater at follow-up compared to post-treatment, and that outcomes will be superior at the second follow-up assessment (12 months) compared to the first (5 months).

## Method

### Trial design

A two-arm randomized controlled trial will be conducted at the Clinical Psychology Unit of Universidad Pontificia Comillas (UNINPSI) in Madrid, Spain. One condition will be based on the EFT model, while the other will be a self-help intervention grounded in EFT and grief theories. This study protocol follows the recommendations of the Standard Protocol Items: Recommendations for Interventional Trials (SPIRIT) (see Additional file 1). Data analysis and reporting will adhere to the guidelines established in the CONSORT (*Consolidated Standards of Reporting Trials*) statement for randomized controlled trials.

The EFT condition will include a total of eight to 10 sessions, depending on the clinician’s judgment, each lasting 60 min and delivered on a weekly basis. Similarly, the self-help intervention will consist of 10 sessions in total, which may extend over approximately 2 to 3 months. An assessment will be conducted prior to the intervention to establish baseline data. In addition, an assessment will take place during the intervention at 3 weeks. Grief and psychological distress will be monitored in all sessions using the Prolonged Grief Scale 13—Revised (PG-13-R) [19] and the Clinical Outcomes in Routine Evaluation-10 (CORE-10) [20], while the therapeutic alliance will be specifically evaluated in sessions 3 and 8 using the Working Alliance Inventory-Short (WAI-S) [21]. Furthermore, another assessment will be conducted at 12 weeks from the start of the intervention (post-treatment), and at 5 and 12 months (follow-up). These assessments, except in the case of the WAI-S, will also be administered in the self-help control group.

Prior to this trial, a pilot study will be conducted with the aim of preventing errors, identifying solutions, and adjusting the trial’s measurement protocol. A total of 22 individuals in individual treatment will voluntarily participate. Compliance and the quality of data collection will be assessed, and working sessions will be organized with the staff to record any difficulties that arise during the process.

### Participants and evaluation

**Fig. 1:**
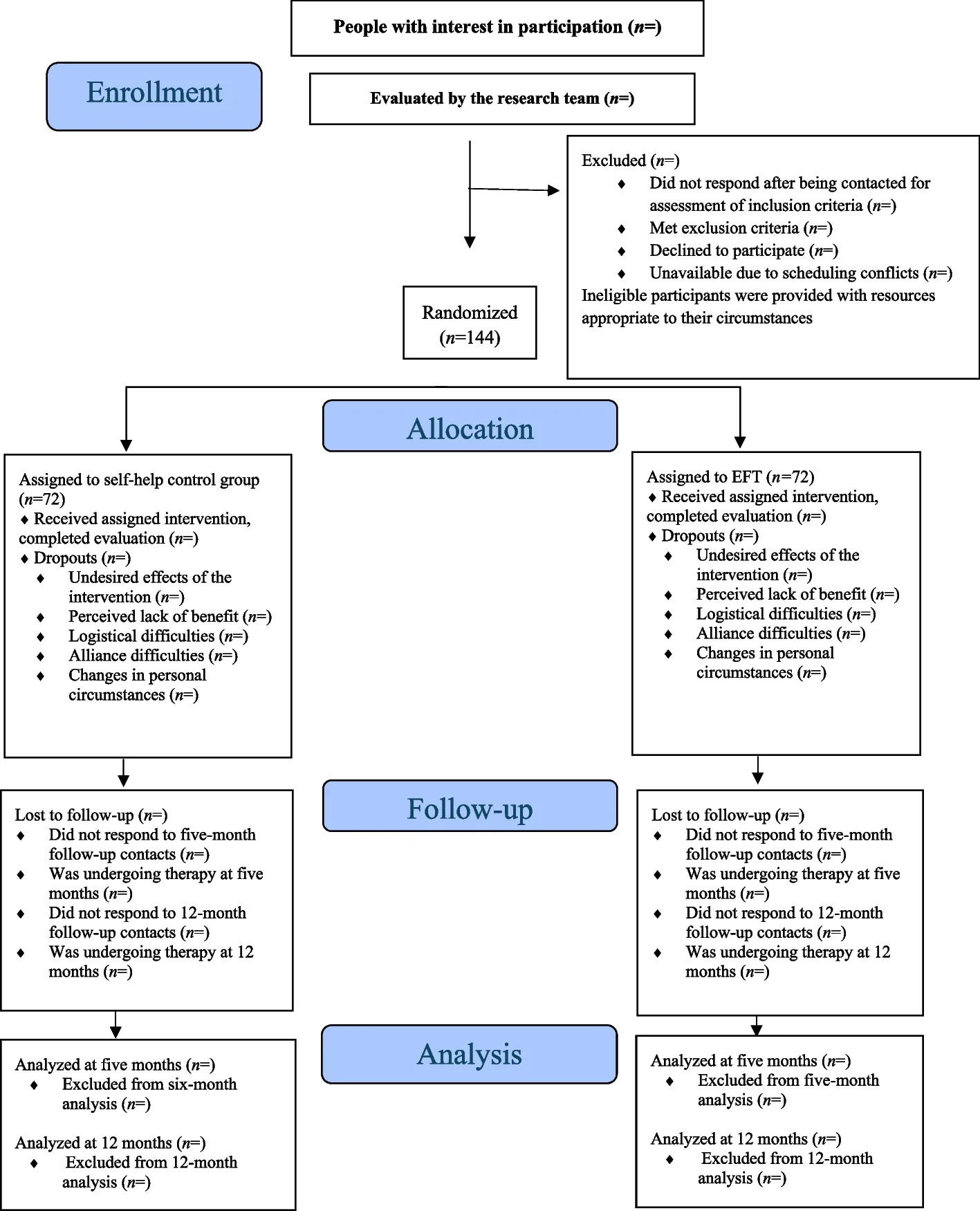
Study flow diagram of referral, screening, and allocation of participants to the EFT for PGD trial

Participants will be recruited through UNINPSI. At the first contact, evaluators will assess the reason for the contact, the time since the death, and the PGD criterion using the PG-13-R [19] and the Traumatic Grief Inventory—Clinician Administered (TGI-CA) [22]. At this stage, initial consent will also be obtained regarding certain characteristics of the study (i.e., audiovisual recording, potential treatment location, use of questionnaires, data protection, and the right to withdraw from the study at any time) in order to proceed with the eligibility assessment. After this, a second, more comprehensive videoconference evaluation will be conducted. In this assessment, eligibility criteria will be evaluated using the Structured Clinical Interview for DSM-5-Research Version (SCID-5-RV) [23], the CORE-10 [20] supplemented with item 6 (“I have been physically violent toward others”) from the Clinical Outcomes in Routine Evaluation–Outcome Measure (CORE-OM) [24], and a clinical interview. If participants meet the eligibility criteria, sociodemographic data will be collected, and the pre-therapy baseline measure will be taken (for participant flow, see Fig. 1). In these cases, trauma-related symptoms will be assessed with the Centrality of Event Scale (CES) [25], psychological distress with the CORE-10, depressive symptoms with the Patient Health Questionnaire-9 (PHQ-9) [26], and anxiety symptoms with the Generalized Anxiety Disorder-7 Scale (GAD-7) [27]. After this assessment, participants will be put in contact with the therapist assigned through the randomization process if they are allocated to the EFT group. If allocated to the self-help control group, they will receive the corresponding self-help exercise each week. Assessments conducted during the intervention will be administered by the therapist while remaining outside the room. Questionnaires will be completed in Microsoft Forms. Before completing the questionnaires, participants will review and sign the informed consent form with the therapist (in the first session). In the case of the self-help control group, these assessments will be carried out via Microsoft Forms at the same time points as in the intervention group, namely at baseline (in this case assessed by videoconference), during the intervention (both at 3 weeks and weekly), and at follow-up.

Participants (*N* = 128) in this clinical trial will be adults (≥18 years) who are undergoing a grief process (including perinatal grief) and who meet the diagnostic criteria for PGD. To determine whether they meet this diagnosis, the death must have occurred 6 months or more prior to the evaluation, and participants must meet the diagnostic criteria for prolonged grief disorder set out in the *International Classification of Diseases, Eleventh Revision* (ICD-11) [28], assessed through the Traumatic Grief Inventory—Clinician Administered (TGI-CA) [22]. Participants must also obtain a score ≥30 on the PG-13-R [19], which sets the clinical threshold and indicates values suggesting a high level of distress [19]. This assessment, together with the fact that it is the person themselves who seeks help, allows these potential participants to be established as a clinical population.

To participate in the study, participants must consent to the research conditions (e.g., pre- and post-therapy assessments and follow-up, focal interview, session videorecording) and confirm this through informed consent. In addition, individuals taking psychopharmacological medication must agree to stabilize with that medication for 6 weeks prior to beginning therapy and, with the approval of their physician, demonstrate their willingness to maintain this stability in medication use throughout the therapy period. Exclusion criteria will include concurrent psychological treatment, bereavement occurring less than 6 months prior to the loss assessed in the PG-13-R, substance abuse, eating disorder, psychosis, and organic brain syndrome (which may be determined during the clinical interview and administration of the SCID-5-RV), as well as suicide risk and risk of harm to others, as defined by scores other than zero on item 16 (“I have made plans to end my life”) and item 6 (“I have been physically violent toward others”) assessed through the CORE-10 with the addition of item 16 from the CORE-OM. In cases where participants meet any of these exclusion criteria, they will be referred to an appropriate service capable of addressing their needs.

**Fig. 2:**
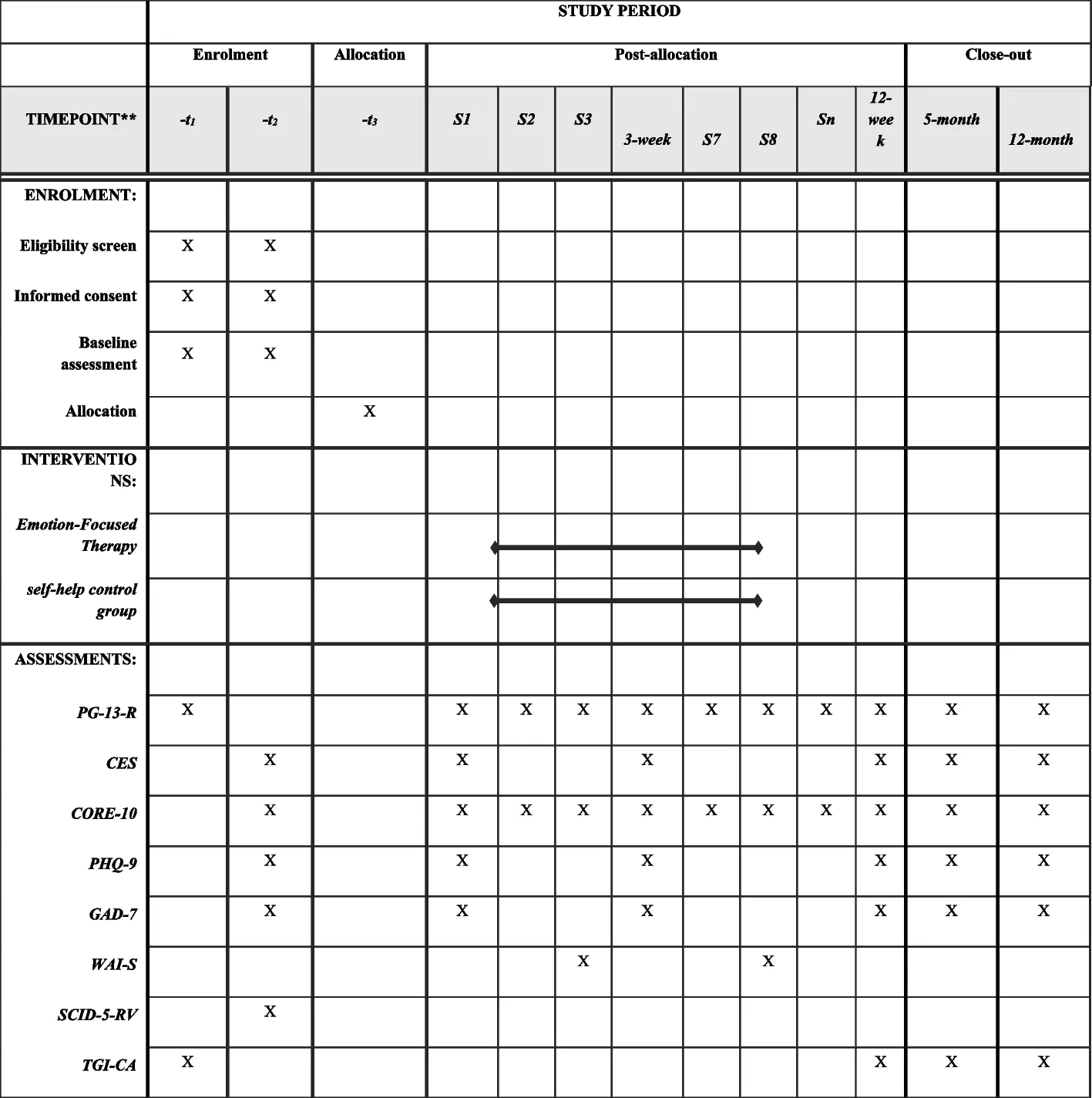
Recommendations for interventional trials (SPIRIT) diagram schedule of enrollment, interventions, and assessments. *PG-13-R* Prolonged Grief Scale 13—Revised, *SCID-5-RV* Structured Clinical Interview for DSM-5-Research Version, *TGI-CA* Traumatic Grief Inventory—Clinician Administered, *CORE-10* Clinical Outcomes in Routine Evaluation-10, *CES* Centrality of Event Scale, *PHQ-9* Patient Health Questionnaire-9, *GAD-7* Generalized Anxiety Disorder-7, *WAI-S* Working Alliance Inventory-Short, -*t1* first assessment, *-t2* second assessment, -*t3* random assignment, *Sn* all sessions

Participants will be randomly assigned with the aim of maintaining balance in the number of participants across conditions. This procedure will be carried out by an independent researcher with no allegiance to either intervention and blinded to the intervention represented by each allocation code. Approximately 1 week after the second assessment, participants will begin sessions aimed at improving symptoms of grief, trauma, anxiety, and depression, in addition to the intervention objectives described in the EFT intervention model [8, 29]. For those assigned to the self-help control group, the intervention will also begin 1 week after the second contact. During the intervention, grief and psychological distress will be assessed in every session (using the PG-13-R and CORE-10), with assessments conducted while the therapist remains outside the room. The therapeutic alliance will also be measured in sessions 3 and 8. In the case of the self-help control group, these assessments will be completed weekly before the corresponding exercise, up to 10 assessments, after which post-treatment and follow-up assessments will be conducted as in the EFT group. Assessments related to dimensions such as depression, anxiety, trauma, psychological distress, and grief symptoms will be conducted at different time points. First, prior to the intervention during the second assessment. Second, 3 weeks after the start of treatment, administered by the participant’s therapist while remaining outside the room, or, in the case of the self-help control group, by a person with no involvement in the research. Third, at the end of the intervention (12 weeks after the start), conducted by an evaluator different from the therapist. Finally, at 5 and 12 months after the start, conducted by an evaluator who is both different from the therapist and blind to the participant’s experimental condition. These assessments will be completed through Microsoft Forms, depending on the participant’s level of competence with this format, except for the TGI-CA, which will be administered via videoconference. The schedule of assessments is illustrated in Fig. 2.

If there are difficulties in contacting the participant at post-treatment or follow-up through the primary means of contact (telephone), alternative channels will be attempted (i.e., email). If the participant has begun another psychotherapeutic intervention, their follow-up data will not be considered (from the moment the intervention has started). On the other hand, if the participant presents intense distress or any kind of clinical difficulty, a comprehensive assessment will be conducted and an appropriate service suited to their circumstances will be provided, with follow-up to determine whether the participant has been able to obtain the necessary help or has encountered any difficulties in doing so.

### Therapists

The clinical trial will involve at least six therapists holding a degree in psychology and the qualifying master’s degree for clinical practice (*Master’s Degree in General Health Psychology*) or clinical licensure (i.e., *Clinical Psychology Resident* [PIR]), who have at least level B accreditation in EFT certified by the International Society of Emotion-Focused Therapy (isEFT). However, additional therapists will be included to anticipate possible dropouts. These therapists will complete a 2-day, 16-h training to receive practical instruction on adapting the EFT model to grief. During this training, they will engage in practice sessions to integrate grief-specific adaptations. In addition, 2-h weekly supervision groups will be held for a period of 3 to 12 months, where therapists will present recordings addressing grief-related themes with their clients. The aim of this process is to ensure stronger adherence to the model. Supervision will continue until sufficient adherence to the EFT model is demonstrated, defined as an average score of four or higher on the nine dimensions of the *Person-Centred and Experiential Psychotherapy Scale: Emotion-Focused Therapy Version* (PCEPS-EFT-9) [30], assessed in two 10-min segments from two consecutive sessions. Once this standard is met, therapists will begin treating participants in the study, continuing in biweekly supervision sessions until the intervention is completed. These supervision and training activities will be conducted by Rafael Jódar, Alejandro de la Traba, and José Gamoneda, in their capacity as accredited EFT trainers and supervisors by isEFT, and based on their practical and research expertise in grief. In addition, adherence during the intervention will be evaluated through the PCEPS-EFT-9 [30]. Similarly, EFT therapists will receive a brief 2-h training session on the recording procedure and will complete a small pilot to identify difficulties and errors. This training will also be carried out by the same supervisors. The therapists will conduct the sessions at the UNINPSI.

### Trial conditions

#### Emotion-focused therapy for grief

The EFT intervention will follow the model described in EFT for grief [8, 29]. The primary objective of this therapy is the transformation of maladaptive primary emotions through the activation of adaptive primary emotions (changing emotion with emotion) [13, 14, 31]. Adaptive primary emotion is understood as the initial response to a given situation, congruent with the situation, and one that, within the therapeutic framework, generates emotional change [13]. Maladaptive primary emotion, in contrast, also arises as an initial reaction to a given situation but is not congruent with it, and is linked to historical pain related to loneliness and abandonment, shame, and fear [14, 32]. To generate this kind of therapeutic change, the aim is to deepen the emotional process, moving from secondary emotions to maladaptive primary emotions, and then articulating unmet needs connected to this core emotional pain [32], which facilitates the emergence of adaptive primary emotions. This process of emotional transformation occurs within a warm and safe therapeutic environment fostered by the therapist’s accepting, empathic, and compassionate stance [14, 32], which seeks attunement to affect and sensitivity to markers (e.g., self-criticism or unfinished business) that indicate suitability for addressing particular affective problems. The process of emotional transformation typically takes place during the enactment of experiential dialogues, which aim to activate emotional pain, whether interpersonal (linked to past unfinished business in significant relationships) or intrapersonal (linked to problematic self-treatments, such as self-criticism), for subsequent transformation within these dialogues. Although this model is transdiagnostic in nature [16], certain specifications can be identified for grief. One such specification is the importance of experiencing the sadness of grief and a sense of connection and presence with the deceased [8]. At the same time, there are commonalities with what has been observed in other difficulties, such as the importance of attending to and fully unfolding maladaptive primary emotions in order to subsequently transform them [8]. For a deeper understanding of the conceptualization of grief processes in EFT, the EFT grief literature can be consulted [8, 29, 33]. This intervention will be carried out at UNINPSI.

In this condition, participants may receive between eight and ten sessions depending on clinical judgment, which will be based on a series of assessments such as (a) the participant continues to present chronic distress, or (b) the participant expresses the need to attend additional sessions, similar to what has been implemented in other clinical trials [32]. In both study conditions, if a participant requires a greater number of sessions, an appropriate service suited to their needs will be offered, unrelated to any financial or other interests connected to the research, its members, or affiliated organizations. If, during or after treatment, situations arise that indicate the suitability of changing the course of treatment (e.g., previously undetected suicide risk, explicit request by the participant), appropriate resources will be sought.

#### Control group: self-help intervention

**Fig. 3:**
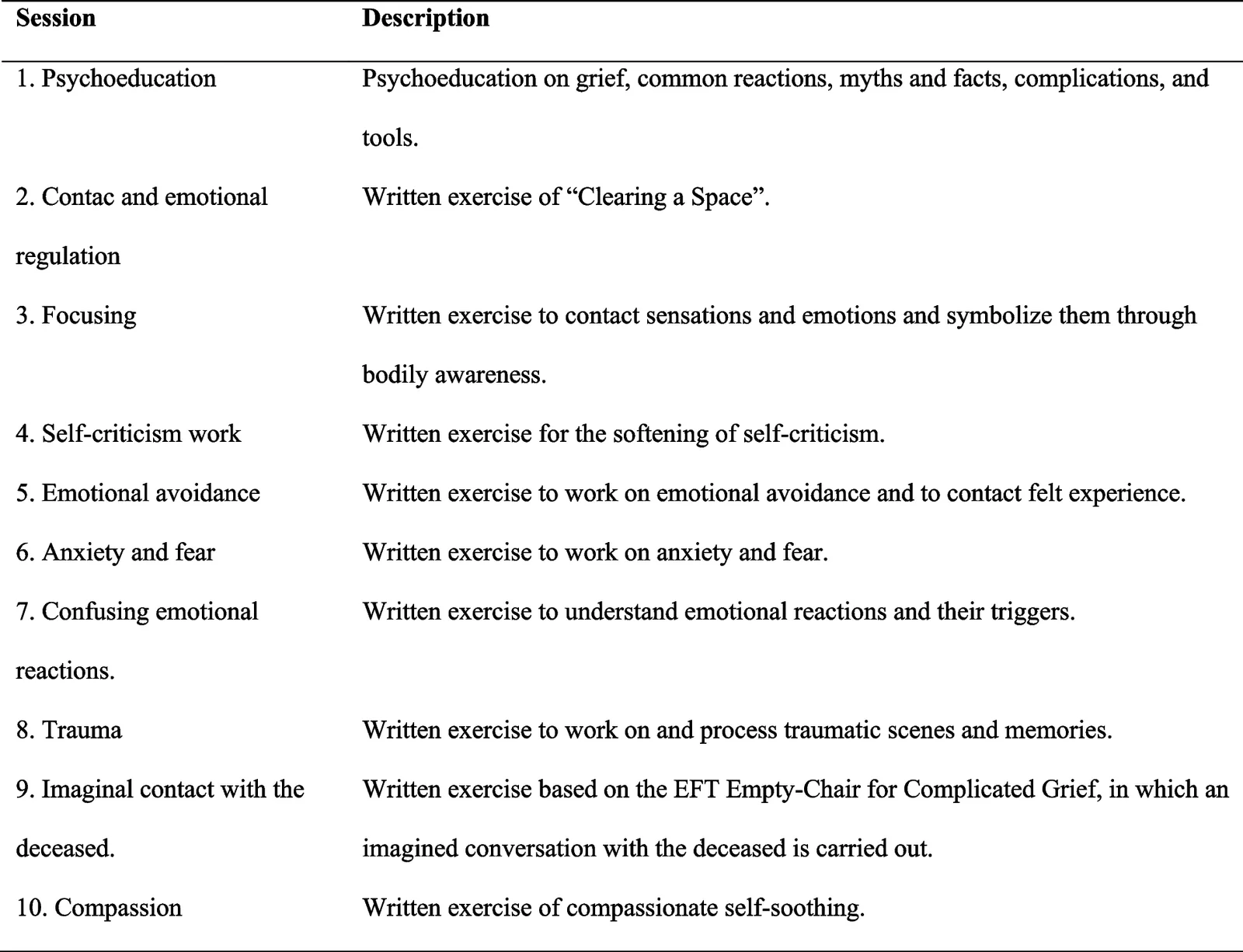
Sessions of a self-help intervention

The control group will consist of 10 sessions of a self-help intervention based on EFT tasks. This intervention is described in Fig. 3.

### Randomization

Participants will be randomly assigned to one of the two study conditions: (1) EFT or (2) self-help control group. Randomization will take place after the second assessment. The allocation sequence will be generated in advance by an independent researcher using permuted block randomization with a single block equal to the total recruitment target (*N* = 144). A vector containing an equal number of allocations to each condition (72 to EFT and 72 to the self-help control group) will be randomly permuted without replacement in R [34] using the sample() function. Each participant identification number (1–144) is thereby assigned to one of the two study conditions. The independent researcher will have access only to the allocation sequence and will remain blinded to the intervention corresponding to each allocation code throughout the allocation process.

The allocation sequence will be accessible only to the independent researcher, who has no allegiance toward either study condition. After participant enrollment, the evaluator will communicate the participant identification number to the independent researcher, who will determine the corresponding allocation and communicate it to the principal investigator. The principal investigator will then inform the participant of the assigned intervention and, if allocated to the EFT condition, will assign the corresponding therapist. To maintain transparency, an auditable record will be generated. Participants will remain unaware of the distinction between the two study conditions, and outcome assessors and data analysts will remain blinded to participants’ group allocation throughout the study, thereby minimizing the risk of assessment and analysis bias.

### Variables and measures

The primary outcome variable will be grief symptomatology, assessed using the PG-13-R. In addition, other outcome variables such as anxiety, depression, trauma, and psychological distress will be assessed, given their relevance and association with grief difficulties [2, 5, 7, 10, 12]. Similarly, therapeutic alliance will be evaluated, along with a series of sociodemographic variables, including age, sex, educational level, primary occupation, quality of social support network, information related to the loss (e.g., time since loss, type of relationship, or whether grief support has been received), and medical information (i.e., use of psychopharmacological medication). Diagnostic status for prolonged grief disorder, assessed with the TGI-CA at baseline, post-treatment, and follow-up, will be analyzed as a secondary dichotomous outcome; all remaining outcome measures are continuous variables.

#### Prolonged grief scale 13—revised (PG-13-R)

The PG-13-R is a revised version of the PG-13 scale adapted to align with the new criteria of the DSM-5-TR [3] for PGD. This instrument consists of 13 items with response options ranging from one (“not at all”) to five (“overwhelmingly”). Validation of the scale [19] demonstrated that grief symptoms represent a unidimensional construct, with high internal consistency indices (Cronbach’s *α* = 0.83, 0.90, and 0.93 across three different samples). Scores of ≥30 indicate the presence of clinical values consistent with grief complications [19].

#### Centrality of event scale (CES)

This instrument measures the centrality of a given experience and is strongly related to complicated grief, post-traumatic stress, and depression [25]. It consists of 20 items with response options ranging from one (“strongly disagree”) to five (“strongly agree”). Prior to completing the scale, participants were instructed to respond with reference to their primary loss. Validation in a Spanish sample yielded Cronbach’s *α* values of 0.92 and 0.94 across two different samples, and a test–retest reliability of *r* = 0.803 [25].

#### Clinical outcomes in routine evaluation–outcome measure (CORE-OM)

This questionnaire assesses psychological distress across the following dimensions: subjective well-being, problems/symptoms, general functioning, and risk. It consists of 34 items scored on a scale ranging from zero to four. Validation in a Spanish sample reported internal consistency (Cronbach’s *α*) ranging from 0.92 to 0.94 across different populations [24]. In the present trial, it will be used for eligibility criteria.

#### Clinical outcomes in routine evaluation-10 (CORE-10)

The CORE-10 measures psychological distress using 10 items selected from the original version for their relevance and coverage of key domains. It shows high internal reliability (Cronbach’s *α* = 0.90) [20]. The clinical cutoff score for general psychological distress is 11, with a reliable change index (90% CI) of six [20]. In the present trial, it will be used both for assessing eligibility criteria and for evaluation after each session.

#### Patient health questionnaire-9 (PHQ-9)

The PHQ-9 is a questionnaire designed to assess the severity of depressive symptoms. It consists of nine items, each scored on a scale from zero (never) to three (nearly every day), with a cutoff score of ≥8 indicating clinical levels of depression. Validation in a Spanish population [26] demonstrated good psychometric properties, with a McDonald’s omega coefficient (*ω*) of 0.84 (95% CI [0.82–0.85]).

#### Generalized anxiety disorder-7 Scale (GAD-7)

The GAD-7 is a unidimensional instrument that measures symptoms of generalized anxiety disorder (GAD). It consists of seven items scored on a scale from zero (never) to three (nearly every day). Validation in a Spanish sample reported a Cronbach’s *α* of 0.93 [27].

#### Working alliance inventory-short (WAI-S)

This instrument comprises three subscales that assess agreement on tasks, agreement on goals, and the therapeutic bond [21]. The WAI-S consists of 12 items, each rated on a seven-point Likert scale ranging from “never” at the lower end to “always” at the upper end. Internal consistency in the Spanish version was high, with a Cronbach’s *α* of 0.91 [21].

#### Structured clinical interview for DSM-5-research version (SCID-5-RV)

This semi-structured diagnostic interview was designed to assess the main DSM-5 diagnoses [23]. In this study, it was used to evaluate exclusion criteria related to psychosis, eating disorders, and substance or alcohol abuse.

#### Traumatic grief inventory—clinician administered (TGI-CA)

The TGI-CA [22] is a 22-item clinician-administered interview designed to establish the diagnoses of prolonged grief disorder defined by the ICD-11 [28] and the DSM-5-TR [3]. Its items are phrased as questions referring to the previous month and are rated on a five-point frequency scale ranging from one (“never”) to five (“always”); a rating of four or five is taken to indicate that the corresponding symptom is present. The instrument provides separate scoring algorithms for the diagnostic criteria of prolonged grief disorder in the ICD-11 [28] and in the DSM-5-TR [3], as well as a total score of disturbed grief severity. In this trial, the ICD-11 criteria will be applied to establish the diagnosis.

Eligibility will be determined by the ICD-11 criteria as assessed with the TGI-CA, requiring at least one criterion B symptom, at least one criterion C symptom, and the criterion E symptom of functional impairment [35], together with the PG-13-R threshold described above. The TGI-CA will be administered at the first assessment to establish eligibility and again at post-treatment and at the 5- and 12-month follow-ups, so that loss of diagnostic status can be examined alongside continuous change in grief severity.

#### Person‐centred and experiential psychotherapy scale: emotion-focused therapy version (PCEPS-EFT-9)

This observational measure is used to assess adherence to the EFT model [30]. It comprises nine dimensions: (1) empathic attunement with the client’s frame of reference/track, (2) accepting presence, (3) emotion focus, (4) dominant or overpowering presence, (5) emotion regulation sensitivity, (6) marker identification, (7) emotion deepening, (8) appropriate use of EFT tasks, and (9) EFT case formulation, defined as the ability to conceptualize clients in EFT terms.

### Assessor training

Prior to this trial, a pilot study will be conducted to examine the feasibility and gather preliminary data on the interventions, as well as to evaluate the data collection process. This will allow potential difficulties to be identified and addressed in preparation for the main trial.

To ensure the quality of the assessment process, therapists who will collect data during the intervention will undergo training and piloting of the assessment procedures. This training will include 1 h dedicated to explaining the data collection process, 1 h for addressing questions, and 1 h for practicing in order to evaluate performance and identify potential difficulties. The training will be conducted by licensed clinical psychologists holding PhD degrees (Rafael Jódar and José Gamoneda). Assessors responsible for data collection at post-treatment and follow-up will remain blinded to participants’ allocation, as they will not be familiar with the intervention process. Their training will consist of 1 h of explanation of the data collection procedures, 1 h for addressing questions, and 2 h of piloting to resolve issues and ensure correct implementation. Individuals conducting the initial videoconference contact to assess eligibility criteria will be specifically instructed in this data collection procedure. Regarding the contact for eligibility assessment and baseline measurement, two assessors with extensive training in administering the study instruments will be involved. Their training will include 4 h of instruction on data collection, 1 to 2 h for addressing questions, and 2 to 3 h of practice to evaluate their performance in administering the measures.

### Sample size

The estimated sample size was 128 participants, equally distributed across the trial conditions. Based on the hypothesis that EFT would have a greater effect than the self-help control group at post-treatment, the sample size calculation indicated the need for 64 participants per condition, resulting in a total sample of *N* = 128. We assumed an effect size (Cohen’s *d*) of 0.5 based on previous evidence from grief interventions [36], with a statistical power of 0.80 and an alpha level of 0.05. Taking into account an estimated dropout rate of 11%, similar to that reported in related studies [37, 38], the required recruitment target was set at 144 bereaved individuals. These sample size calculations were determined using G*Power [39].

Recruitment will be supported through UNINPSI, advertisements, and collaboration with healthcare services. These strategies are expected to ensure achievement of the target sample size over the planned 2-year recruitment period.

### Data management

Data will be collected, coded, and stored in anonymized form, with participants identified by a code (which will also identify the therapist), and will not contain any personally identifiable information. Coding and data storage will be safeguarded using secure, password-protected electronic databases accessible only to authorized members of the research team. A control list allowing identification and verification of research data and procedures, together with regular backups and auditable records, will be maintained by the principal investigator in accordance with institutional data protection policies. Adverse events will be documented by the principal investigator, and any related decisions will be made by the research team.

Data will be periodically exported in formats compatible with statistical software, ensuring compliance with ethical and data protection standards, maintaining auditable records, and safeguarding participant confidentiality. The final dataset will be provided in CSV format, accompanied by a codebook describing each variable and its possible values, under the custody of the principal investigator.

Paper records will be coded to prevent identification and safeguarded in a locked cabinet. Video recordings will be encrypted and stored on a hard drive kept in a locked cabinet. Quantitative data may be shared upon reasonable request by any interested researcher, while access to qualitative data will be restricted and requests will be reviewed on a case-by-case basis. Access to recordings and transcripts, given the greater challenges of anonymization, will also be managed individually by the responsible investigator. The project has been approved by the Ethics Committee of Universidad Pontificia Comillas. Any data made publicly accessible will be fully anonymized to prevent participant identification. Research data will be deleted 10 years after the last follow-up assessment.

### Statistical methods

Analyses will be conducted under the supervision of Pablo F. Cáncer. To compare change in the primary outcome variables (PG-13-R and CORE-10) and secondary outcome variables (GAD-7, PHQ-9, and CES) across the assessment points (baseline, 3 weeks, and 5 and 12 months) and between the two intervention groups, linear mixed models will be employed. Effect sizes for both intervention groups will be calculated using Cohen’s *d*, along with the differential effect between conditions. In addition, analyses will be performed to examine the proportion of participants achieving clinically significant change, recovery rates (clinically significant change combined with a shift to non-clinical ranges), and reliable deterioration at post-treatment and follow-up with respect to symptoms of grief complications. The proportion of participants no longer meeting ICD-11 criteria for prolonged grief disorder on the TGI-CA at post-treatment and follow-up will be compared between conditions using generalized linear mixed models with a logit link, on an intention-to-treat basis. Remission will be defined by the same criteria used to establish eligibility [35], so that all participants meet criteria at baseline by definition; change under the more conservative rule [40] will be reported as a sensitivity analysis in the subgroup meeting that rule at baseline.

These analyses will be conducted both for study completers (those who attended between eight and ten sessions) and on an intention-to-treat basis, including all participants initially assigned to the intervention groups regardless of their status at study completion. For the latter, mixed-effects models will be estimated using full information maximum likelihood (FIML) with all available data. Moderation analyses will examine whether baseline grief severity and therapeutic alliance are associated with treatment outcomes. Changes in grief symptoms will also be examined in relation to concomitant changes in psychological distress, depression, and anxiety, given their intertwined associations [41]. These analyses will be considered exploratory.

The data analyst will be blinded to participants’ group allocation. Data analyses will be conducted using R [34], with a confidence level set at 95%.

### Governance and oversight of the trial

The trial coordination team will meet monthly for general project oversight and will be composed of José Gamoneda and Rafael Jódar. José Gamoneda will oversee the supervision of the EFT intervention and data collection for both the EFT and self-help control groups. Rafael Jódar and Pablo F. Cáncer will be responsible for supervising the data analyses, while José Gamoneda will also act as the supervisor of training and clinical supervision. A consortium will be established for the overall evaluation of the trial, consisting of the PI, EFT trainer and supervisor (JG), the researcher, EFT trainer and supervisor (RJ), and one of the supervisors of data analysis (PFC). This coordination team will address issues such as protocol design and implementation, adherence to the interventions, and various matters related to participants and the interventions, as well as any difficulties or adverse events that may arise. Any substantial modification of the protocol will be decided by the coordination team and communicated to the investigators, the ethics committee, the trial registry, and, if applicable, to participants and other relevant stakeholders. The team will also collaborate with JMC, as an expert in research methodology, to provide support and guidance on methodological and other challenges related to the trial. No independent external auditing of trial conduct is planned. Oversight of the study will instead be provided through the trial coordination team, which will review study conduct, protocol adherence, recruitment, data quality, adverse events, and protocol deviations during its monthly meetings.

## Expected results

We hypothesize that the EFT condition may demonstrate greater improvements than the self-help control group across the intervention, at post-treatment, and at follow-up assessments. However, these expected differences are tentative, as the evidence for EFT in the treatment of prolonged grief disorder is still preliminary and no randomized controlled trial has yet evaluated this model for PGD. The present study is therefore designed to provide an initial, rigorous test of its comparative efficacy.

## Discussion

There is strong evidence supporting cognitive-behavioral treatments for PGD, as demonstrated by several studies [42]. However, other commonly used treatments for this condition still lack sufficient empirical support, despite some initial evidence [8]. Given their use in practice and the recognized need for greater heterogeneity in grief interventions [7], this trial aims to test the efficacy of EFT. The purpose is to examine whether EFT shows promise as an effective treatment for PGD and to provide preliminary evidence through a rigorous randomized controlled design. This aims to provide an alternative for those bereaved individuals who do not benefit from the approaches tested thus far [7]. This is understood as a necessary response to suffering that is not yet being addressed effectively, a concern that becomes even more pressing in light of the high numbers of individuals with grief complications, particularly since COVID-19.

### Limitations

The proposed study entails several limitations. One of them is inherent to psychotherapy trials, namely the challenge of ensuring adequate blinding, as achieving full participant blinding is considerably more difficult in psychotherapy research. In addition, the adjusted sample size and potential attrition during follow-up may constitute a future limitation. Nonetheless, this has been addressed by planning a sufficiently large sample and by accounting for expected participant loss throughout the research process. Finally, therapist-administered assessments may introduce a degree of bias. However, efforts have been made to mitigate this through an evaluation protocol requiring therapists to remain outside the session room.

## Supplementary Information

Additional file 1: SPIRIT Outcomes 2022 Checklist with SPIRIT 2013: recommended items of a clinical trial protocol.

Additional file 2: Informed consent.

Additional file 3: Original approval by the Ethics Committee.

Additional file 4: English version of approval by the Ethics Committee.

## Data Availability

Data are not currently available, as the trial has not yet been completed. Once the results are published, relevant data and materials will be made available upon reasonable request to the corresponding author. Due to the challenges of ensuring confidentiality in qualitative information, requests related to these data will be reviewed on a case-by-case basis.

## Abbreviations

APA: American Psychiatric Association
CES: Centrality of Event Scale
CONSORT: Consolidated Standards of Reporting Trials
CORE-10: Clinical Outcomes in Routine Evaluation-10
CORE-OM: Clinical Outcomes in Routine Evaluation–Outcome Measure
DSM-5-TR: 5Th edition of the Diagnostic and Statistical Manual of Mental Disorders
EFT: Emotion-focused therapy
GAD-7: Generalized Anxiety Disorder-7 Scale
ICD-11: International Classification of Diseases, Eleventh Revision
INE: Instituto Nacional de Estadística de España [Spanish National Institute of Statics]
isEFT: International Society of Emotion-Focused Therapy
PCEPS-EFT-9: Person‐Centred and Experiential Psychotherapy Scale: Emotion-Focused Therapy Version
PG-13-R: Prolonged Grief Scale 13—Revised
PGD: Prolonged grief disorder
PHQ-9: Patient Health Questionnaire-9
RCI: Reliable change index
RCT: Randomized controlled trial
SCID-5-RV: Structured Clinical Interview for DSM-5-Research Version
SPIRIT: Standard Protocol Items: Recommendations for Interventional Trials
UNINPSI: Unidad Clínica de Psicología de la Universidad Pontificia Comillas [Psychological Clinic Unit of the Pontifical Comillas University]
TGI-CA: Traumatic Grief Inventory—Clinician Administered
WAI-S: Working Alliance Inventory-Short
