# Supplementary material for: Emotion-focused therapy in the treatment of prolonged grief disorder: study protocol for a randomized controlled trial
Source: Trials. 2026 Sep 21;27:589. doi: 10.1186/s13063-026-10005-0 (PMC13625471; doi:10.1186/s13063-026-10005-0)
Supplement: Supplementary file 1 [file 13063_2026_10005_MOESM3_ESM.pdf]

COMILLAS
UNIVERSIDAD PONTIFICIA
ICAI ICADE CIHS

Madrid, 30 de octubre de 2025

Dictamen 018/25-26

Para: Excmo. Vicerrector de Profesorado, Investigación e Inteligencia Artificial

Asunto: Juicio del Comité de Ética acerca del proyecto titulado: "ENSAYO CLÍNICO ALEATORIZADO DE TERAPIA FOCALIZADA EN LA EMOCIÓN (TFE) EN TRASTORNO DE DUELO PROLONGADO (PGD)", presentado por el Profesor D. José Gamoneda Larripa de la Facultad de Ciencias Humanas y Sociales.

El Comité de Ética de la Investigación de la Universidad Pontificia Comillas, conforme al procedimiento establecido, habiendo sido valorado el caso por sus miembros, emite el siguiente DICTAMEN:

El proyecto salvaguarda la dignidad de las personas participantes en la investigación. Estas son hombres y mujeres mayores de edad que sufren duelo prolongado a causa del fallecimiento de un ser querido. El proyecto está bien justificado y sus objetivos resultan delimitados. Con motivo de la investigación los participantes podrán mejorar sus síntomas y obtener un mayor bienestar emocional. No existen riesgos sustanciales para los participantes, y si fuera necesario contarán con apoyo psicológico. La investigación no se efectúa con menores de edad. Todas las preguntas que se formulan resultan pertinentes. Por tanto, el proyecto de investigación es conforme con los principios de la Declaración de Helsinki, en cuanto resultan mayores sus beneficios que sus riesgos.

Los participantes en el proyecto gozan de autonomía para determinar si intervienen o no en el mismo, siendo también informados de que en cualquier momento pueden retirar su consentimiento para participar sin necesidad de dar ninguna explicación y sin temer ninguna represalia. El investigador se compromete a guardar confidencialidad y anonimicidad en la recogida de datos del estudio, de forma que la identificación de los participantes no resulte posible. Las grabaciones que se efectúen en la investigación serán realizadas conforme a los criterios de la Ley Orgánica 1/1982 de protección del derecho al honor, la propia imagen y la intimidad personal y familiar; y dichas grabaciones únicamente serán utilizadas por el equipo investigador. La investigación es conforme con la Ley Orgánica 3/2018 de Protección de Datos Personales y demás legislación concordante.

El proyecto merece un juicio de conformidad ética para una investigación de sus características, y cuenta con la aprobación de este Comité.

Atentamente,

Dr. Miguel Grande Yáñez
Presidente

Raúl González

Dr. Raúl González Fabre
Secretario
