# Supplementary material for: Emotion-focused therapy in the treatment of prolonged grief disorder: study protocol for a randomized controlled trial
Source: Trials. 2026 Sep 21;27:589. doi: 10.1186/s13063-026-10005-0 (PMC13625471; doi:10.1186/s13063-026-10005-0)
Supplement: Supplementary file 3 [file 13063_2026_10005_MOESM2_ESM.docx]

1. **ORIGINAL INFORMED CONSENT (SPANISH)**

**Consentimiento informado usuario**

Título del Proyecto de Investigación: *Estudio de Terapia Focalizada en la Emoción (TFE) en duelo*

Institución responsable de la investigación: Universidad Pontificia Comillas.

**DECLARA QUE HA SIDO INFORMADO/A QUE:**

PARTICIPACIÓN VOLUNTARIA

Debe saber que su participación en este estudio es voluntaria y que puede decidir no participar o cambiar su decisión y retirar el consentimiento en cualquier momento, sin necesidad de explicar sus motivos y sin que exista ningún tipo de repercusión negativa por su decisión.

DESCRIPCIÓN GENERAL DEL ESTUDIO

Este estudio busca evaluar la efectividad de la intervención ante el duelo desde el modelo humanista. Para ello se le administrarán cuestionarios en diferentes momentos del proceso de acompañamiento, para evaluar la evolución de las dificultades psicológicas. El tiempo para contestar estos cuestionarios varía entre 5 y 15 minutos. Estos cuestionarios se contestarán por vía telefónica, en papel o a través de Microsoft Forms.

BENEFICIOS Y RIESGOS DERIVADOS DE SU PARTICIPACIÓN EN EL ESTUDIO

Beneficios:

Su participación le podría generar un mayor grado de conciencia y conocimiento de sus síntomas y procesos emocionales. Este autoconocimiento podría contribuir positivamente al acompañamiento que recibe. Su participación permitirá entender la magnitud del cambio y los tipos de síntomas que cambian tras la intervención en duelo. Su participación podría suponer una reducción del malestar y un mayor bienestar emocional, como resultado del proceso terapéutico.

Riesgos:

Contestar estos cuestionarios puede hacerse tedioso en algún momento del proceso para algunas personas. Algunas de las preguntas pueden traerte temas o relaciones a la imaginación con el malestar emocional asociado. Para minimizar estos riesgos, hemos seleccionado cuestionarios breves. Los terapeutas y personas de apoyo han sido formados para estar atentos a cualquier reacción adversa que puedas experimentar en el proceso de rellenar estos cuestionarios. Puedes contactar con José Gamoneda (jgamoneda@comillas.edu) para futuras dudas.

CONFIDENCIALIDAD Y RESPONSABILIDADES

El estudio se realizará bajo las indicaciones de la Declaración de Helsinki y según la legislación española vigente:

- Ley 14/2007, de 3 de julio, de Investigación biomédica (BOE núm. 159, de 4 de julio de 2007).
- Orden SSI/81/2017, de 19 de enero (BOE núm. 31, de 6 de febrero de 2017) por la que se publica el Acuerdo de la Comisión de Recursos Humanos del Sistema Nacional de Salud, por el que se aprueba el protocolo mediante el que se determinan pautas básicas destinadas a asegurar y proteger el derecho a la intimidad del paciente por los alumnos y residentes en Ciencias de la Salud.

Todos los datos recogidos para el estudio estarán identificados mediante un código y en ningún caso podrán relacionarse con usted. Los investigadores se comprometen a custodiar los datos de acuerdo con la Ley Orgánica 3/2018, de 5 de diciembre, de Protección de Datos Personales y garantía de los derechos digitales (LOPDGDD) y el Reglamento 2016/679 del Parlamento Europeo y del Consejo de 27 de abril de 2016 relativo a la protección de las personas físicas en lo que respecta al tratamiento de datos personales y a la libre circulación de estos datos. Las personas legitimadas podrán ejercer el derecho de acceso, rectificación, cancelación, oposición, olvido, limitación del tratamiento, regulados en la LOPDGDD y en los establecido en el Reglamento Europeo y del Consejo de 27 abril de 2016 relativo a la protección de las personas físicas en lo que respecta al tratamiento de datos personales y a la libre circulación de estos datos y demás normativa de desarrollo, así́ como a las normas que en el futuro puedan ampliarlas o sustituirlas. El acceso a su información personal quedará restringido al equipo investigador del estudio cuando lo precisen para comprobar los datos y procedimientos del estudio, pero siempre manteniendo la confidencialidad de estos de acuerdo con la legislación vigente. Los resultados y datos obtenidos en la investigación serán borrados a los 10 años de la finalización de la última evaluación realizada por el equipo de investigación (12 meses desde el inicio de la investigación).

Los resultados de este estudio podrán ser difundidos en congresos o revistas científicas, siempre de forma anónima y sin que sea posible identificar a ningún participante.

COMPENSACIÓN ECONÓMICA

Su participación en el estudio no le supondrá ningún gasto y tampoco conllevará ningún tipo de compensación económica.

OTRA INFORMACIÓN RELEVANTE

Si usted decide retirar el consentimiento para participar en este estudio, ningún dato nuevo será añadido.

***Eficacia de la intervención humanista en duelo***

Yo,……………………………………………………………………………………………… (Nombre, apellido)

- He leído la hoja de información previa
- He podido hacer todas las preguntas que he considerado sobre el estudio.
- He recibido respuestas satisfactorias a mis preguntas.
- He recibido suficiente información sobre el estudio.
- He sido informado de que puedo hablar con José Gamoneda (jgamoneda@comillas.edu) para futuras dudas.
- Comprendo que mi participación es voluntaria.
- Comprendo que puedo retirarme del estudio:
  - Cuando quiera.
  - Sin tener que dar explicaciones.
  - Sin que esto repercuta en mi proceso de acompañamiento.
- Presto libremente mi conformidad para participar en el estudio.
- Declaro que he comprendido la información proporcionada y acepto participar en este estudio.
- Doy mi consentimiento a que las grabaciones y los cuestionarios sean utilizados para los siguientes fines:
- La investigación, preservando su anonimato. Sí  No 
- La docencia y formación de psicólogos dentro del centro. Sí  No 
- En entrenamiento de otros profesionales de la salud mental fuera del centro, (seminarios de trabajo, conferencias profesionales). Sí  No 
- Autorizo el uso de la información proporcionada en el marco de esta investigación para su inclusión, de forma anónima y confidencial, en publicaciones académicas y/o libros relacionados con el estudio. Sí  No 

Fecha: ……………………………….

Nombre y apellidos: ……………………………….

DNI: ……………………………….

**Firma:**

1. **INFORMED CONSENT (ENGLISH)**

Research Project Title: *Emotion-Focused Therapy (EFT) Study on Grief*

Responsible Institution: Pontifical Comillas University

**INFORMED CONSENT FORM**

**VOLUNTARY PARTICIPATION**

You should know that your participation in this study is voluntary. You may decide not to participate or to withdraw your consent at any time, without having to give reasons and without any negative consequences for your decision.

**GENERAL DESCRIPTION OF THE STUDY**

This study aims to evaluate the effectiveness of an intervention for grief based on the humanistic model. To this end, you will be asked to complete questionnaires at different points during the accompaniment process, to assess changes in psychological difficulties over time. Completing these questionnaires will take between 5 and 15 minutes. They may be completed by phone, on paper, or via Microsoft Forms.

**BENEFITS AND RISKS OF PARTICIPATION**

Benefits:
Your participation may help you gain greater awareness and understanding of your symptoms and emotional processes. This self-knowledge may contribute positively to your therapeutic process. Your participation will also help researchers understand the extent of change and the types of symptoms that improve following a grief intervention. Participation may also lead to a reduction in distress and greater emotional well-being as a result of therapy.

Risks:
For some participants, completing the questionnaires may feel tedious at times. Some questions may evoke distressing memories or emotions. To minimize these risks, we have selected brief questionnaires. Therapists and support staff have been trained to monitor any adverse reactions you may experience while completing the questionnaires. You may contact José Gamoneda (jgamoneda@comillas.edu) if you have any questions or concerns.

**CONFIDENTIALITY AND RESPONSIBILITIES**

The study will be conducted in accordance with the principles of the Declaration of Helsinki and current Spanish legislation:

- Law 14/2007, of July 3, on Biomedical Research (BOE No. 159, July 4, 2007).
- Order SSI/81/2017, of January 19 (BOE No. 31, February 6, 2017), which publishes the Agreement of the Human Resources Commission of the National Health System approving the protocol for ensuring and protecting patients’ right to privacy in interactions with students and residents in Health Sciences.

All data collected for the study will be identified by a code and will not be linked to your personal identity. The researchers commit to handling all data in accordance with Organic Law 3/2018, of December 5, on the Protection of Personal Data and the Guarantee of Digital Rights (LOPDGDD), and Regulation (EU) 2016/679 of the European Parliament and of the Council of April 27, 2016 (GDPR).

Participants may exercise their rights of access, rectification, cancellation, opposition, erasure (“right to be forgotten”), and limitation of processing as regulated in the LOPDGDD and GDPR, as well as in any future laws that may expand or replace them.

Access to your personal information will be restricted to the research team only when necessary for verification of study data and procedures, always maintaining confidentiality in accordance with applicable legislation.

All study data and results will be deleted 10 years after completion of the final participant assessment (12 months after study initiation).

The results of this study may be presented at conferences or published in scientific journals, always anonymously and without any possibility of identifying participants.

**FINANCIAL COMPENSATION**

Your participation will not involve any financial cost or compensation.

**ADDITIONAL INFORMATION**

If you decide to withdraw your consent, no new data will be collected from you after that point.

Effectiveness of the Humanistic Intervention in Grief

I, ......................................................................................... (Name, Surname)

- Have read the information sheet provided.
- Have had the opportunity to ask all questions I considered necessary about the study.
- Have received satisfactory answers to my questions.
- Have received sufficient information about the study.
- Have been informed that I can contact José Gamoneda (jgamoneda@comillas.edu) for any further questions.
- Understand that my participation is voluntary.
- Understand that I may withdraw from the study:
  - Whenever I wish.
  - Without having to give explanations.
  - Without this affecting my therapeutic process.
- Freely give my consent to participate in the study.
- Declare that I have understood the information provided and agree to participate in this study.
- Give my consent for the recordings and questionnaires to be used for the following purposes:
  - For research purposes, ensuring anonymity. Yes ☐ No ☐
  - For teaching and training psychology students within the center. Yes ☐ No ☐
  - For training other mental health professionals outside the center (work seminars, professional conferences). Yes ☐ No ☐
  - I authorize the anonymous and confidential use of the information provided for inclusion in academic publications and/or books related to this study. Yes ☐ No ☐

Date: ..............................................
Full name: ..............................................
ID (DNI): ..............................................
Signature: ..............................................
