# Supplementary material for: Emotion-focused therapy in the treatment of prolonged grief disorder: study protocol for a randomized controlled trial
Source: Trials. 2026 Sep 21;27:589. doi: 10.1186/s13063-026-10005-0 (PMC13625471; doi:10.1186/s13063-026-10005-0)
Supplement: Supplementary file 4 [file 13063_2026_10005_MOESM4_ESM.docx]

**Madrid, October 30, 2025**
**Report No. 018/25-26**

To: The Vice-Rector for Faculty, Research, and Artificial Intelligence

**Subject:** Ethical Review of the Research Project entitled:
*“Randomized Controlled Trial of Emotion-Focused Therapy (EFT) for Prolonged Grief Disorder (PGD)”*,
submitted by Professor Dr. José Gamoneda Larripa, Faculty of Human and Social Sciences.

The Research Ethics Committee of Pontifical Comillas University, following the established procedures and after thorough evaluation by its members, issues the following statement:

The project safeguards the dignity of the individuals participating in the study. Participants are adult men and women who experience prolonged grief following the death of a loved one. The project is well-justified, and its objectives are clearly defined. Through participation in the study, participants are expected to improve their symptoms and achieve greater emotional well-being. There are no substantial risks for participants, and psychological support will be available if necessary. The study does not involve minors, and all questions included are considered appropriate. Therefore, the research project complies with the principles of the Declaration of Helsinki, as the potential benefits outweigh the possible risks.

Participants in this project retain full autonomy to decide whether to participate. They have been informed that they may withdraw their consent at any time without explanation and without any negative consequences. The principal investigator is committed to maintaining confidentiality and anonymity in data collection, ensuring that participants cannot be personally identified. Any recordings made as part of the research will adhere to the principles established in Organic Law 1/1982 on the protection of the right to honor, personal and family privacy, and one’s own image. These recordings will be used exclusively by the research team. The study also complies with Organic Law 3/2018 on the Protection of Personal Data and Guarantee of Digital Rights, as well as other applicable legislation.

**The Committee considers that this project meets the ethical standards required for research of its nature and hereby grants its approval.**

Sincerely,

**Dr. Miguel Grande Yáñez**
**Dr. Raúl González Fabre**

Pontifical Comillas University
Research Ethics Committee
